# Underlying Features of Prostate Cancer—Statistics, Risk Factors, and Emerging Methods for Its Diagnosis

**DOI:** 10.3390/curroncol30020178

**Published:** 2023-02-15

**Authors:** Cristina V. Berenguer, Ferdinando Pereira, José S. Câmara, Jorge A. M. Pereira

**Affiliations:** 1 CQM—Centro de Química da Madeira, NPRG, Campus da Penteada, Universidade da Madeira, 9020-105 Funchal, Portugal; 2 SESARAM—Serviço de Saúde da Região Autónoma da Madeira, EPERAM, Hospital Dr. Nélio Mendonça, Avenida Luís de Camões 6180, 9000-177 Funchal, Portugal; 3 Departamento de Química, Faculdade de Ciências Exatas e Engenharia, Campus da Penteada, Universidade da Madeira, 9020-105 Funchal, Portugal

**Keywords:** prostate cancer, incidence, mortality, risk factors, biomarkers

## Abstract

Prostate cancer (PCa) is the most frequently occurring type of malignant tumor and a leading cause of oncological death in men. PCa is very heterogeneous in terms of grade, phenotypes, and genetics, displaying complex features. This tumor often has indolent growth, not compromising the patient’s quality of life, while its more aggressive forms can manifest rapid growth with progression to adjacent organs and spread to lymph nodes and bones. Nevertheless, the overtreatment of PCa patients leads to important physical, mental, and economic burdens, which can be avoided with careful monitoring. Early detection, even in the cases of locally advanced and metastatic tumors, provides a higher chance of cure, and patients can thus go through less aggressive treatments with fewer side effects. Furthermore, it is important to offer knowledge about how modifiable risk factors can be an effective method for reducing cancer risk. Innovations in PCa diagnostics and therapy are still required to overcome some of the limitations of the current screening techniques, in terms of specificity and sensitivity. In this context, this review provides a brief overview of PCa statistics, reporting its incidence and mortality rates worldwide, risk factors, and emerging screening strategies.

## 1. Introduction

Prostate cancer (PCa) is the second most frequent type of malignancy cancer among men worldwide [1,2]. PCa burden was very dramatic until the beginning of the 21st century, due to the increased use of the prostate-specific antigen (PSA) tests for screening. From this date onwards, different innovations increasing the efficacy of the therapeutic methods, along with earlier diagnoses, led to a significant reduction in the number of deaths, and a less pronounced downward trend in the incidence of PCa.

Epidemiological studies have shown that the geographical and racial distribution differences in PCa incidence and mortality rates reflect differences in the distribution of populations, with varying degrees of genetic susceptibility [3,4]. Epigenetic factors such as different lifestyles also contribute to these differences, particularly unbalanced diets, and tobacco and alcohol consumption [2,3,5]. Another difference is in the availability and use of, and access to, medical care, especially regional differences in the diagnosis of latent cancers through PSA screening [5,6]. Generally, most men are reluctant to go through PCa screening, since it is based on invasive and unpleasant procedures. For cancer control, it is of the utmost importance to build a sustainable platform for the dissemination of cancer prevention and the provision of cancer care, specifically in low-income and transitioning countries. These results highlight the need to increase health literacy and ensure that opportunistic screening is preceded by a thorough discussion about its potential benefits and risks [7]. Hence, it is crucial to develop more focused diagnostic tools for the early and non-invasive detection of PCa that can classify patients according to the severity of their cancers, and, as a result, guide their treatment decisions. In this review, PCa statistics are briefly summarized, reporting its incidence and mortality rates worldwide, and risk factors and emerging screening strategies are presented and discussed.

## 2. Incidence and Mortality Rates Worldwide

**Figure 1:**
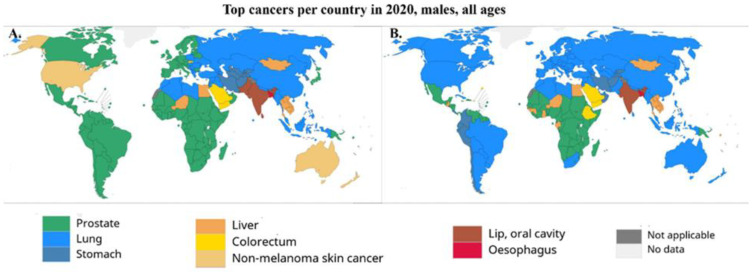
(**A**) Most diagnosed types of cancer among men worldwide, 2020. Nonmelanoma skin cancer was included in calculations of top cancer per country. (**B**) Leading cause of cancer deaths among men worldwide, 2020. Source: GLOBOCAN 2020 [1].

The prevalence of PCa varies among different racial groups, and the vast disparity has been associated with socioeconomic conditions, as well as environmental and biological factors, which play an important role in the etiology of PCa. Variations in the incidence rates may be due to underdiagnosis, differences in screening methods, and disparities in healthcare access [2]. Requesting PSA tests directly influences the incidence values around the world. In more developed countries, the use of the PSA test has resulted in a reduction in the mortality rates, while in less developed countries, they have shown an increase, reflecting the access to early detection and available therapies yielded by the PSA result [1,8]. For instance, PCa incidence in Europe is high when compared with other geographical areas, such as Africa or Asia, due to the use of PSA for early detection [9]. Regional differences are related to environmental risk factors and differences in healthcare policies across individual countries, such as the access to and availability of costly targeted therapies, in addition to heterogeneity in health and socioeconomic status [9,10]. In 2020, PCa was the most frequently diagnosed cancer among men in 121 of 185 countries around the world [1,2,5] (Figure 1). The world age-standardized incidence rates (wASR) are three times higher in areas with high or very high human development index scores [1,5] when compared with less developed countries (37.5 and 11.3/100,000, respectively), while the mortality rates are almost constant (8.1 and 5.9/100,000, respectively).

Social determinants, such as poverty, lack of education, lack of social support, and social isolation, play an important role in the PCa stage at diagnosis and survival. A later stage at diagnosis may be due to lower PCa screening rates or population-specific variations in environmental exposures, including diet, physical activity, or occupational exposures. Additionally, men may be persuaded by their partner, other family members, or others within their social network to undergo PCa screening [11]. Social media can be employed in research, advocacy, and awareness campaigns in the PCa community. Evidence suggests that social media initiatives may enhance cancer screening and early detection. Patients and their caregivers can also take advantage of networking and educational opportunities. Nevertheless, a few concerns remain regarding inconsistent information quality [12].

**Figure 2:**
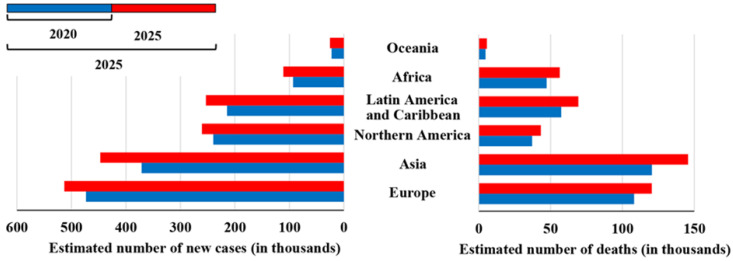
Estimated number of new cases and deaths from prostate cancer from 2020 to 2025. Source: GLOBOCAN 2020 [1,5].

Overall, in the last 5 years, the mortality rates have declined, most probably due to improved access to treatments and dissemination of therapies, such as surgery and hormonotherapy. The projections for the next 5 years show an increasing trend in the estimated number of new cases and deaths (Figure 2), for all continents. Furthermore, in the upcoming years, the number of PCa cases may increase, because the diversion of resources to the COVID-19 pandemic has delayed diagnosis, patient management, treatment, and research. Many cancer patients had their management delayed as PCa care changed and shifted towards patterns that limited the risk of COVID-19 infection, including increased use of transperineal biopsy and hypofractionated radiation therapy regimens, as well as the substitution of docetaxel with enzalutamide [13]. This pandemic will lead to an increasing number of men diagnosed with more advanced diseases, which will have a negative impact on their prognosis. Consequently, treating patients with locally advanced or metastatic diseases is also expected to be more expensive than treating those with less advanced diseases. Therefore, to control the clinical, economical, and welfare costs to society, urgently coordinated action is needed to address the diagnostic and treatment deficiencies in PCa services [13].

## 3. Prostate Cancer Risk Factors

**Figure 3:**
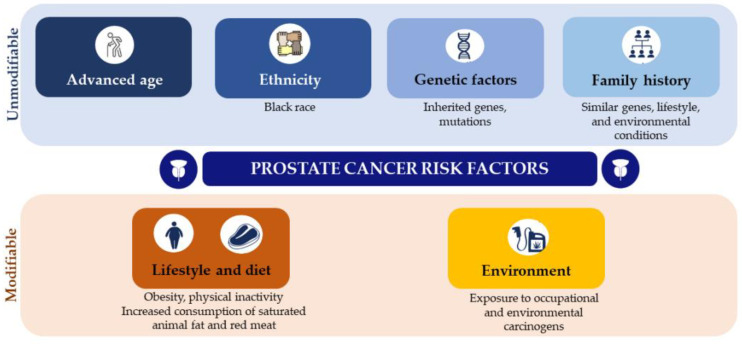
Modifiable and unmodifiable prostate cancer risk factors.

The well-established PCa risk factors are advancing age, ethnicity (Black race), certain genetic mutations, insulin-like growth factors (IGF), and family history of this malignancy (Table 1) [5]. Lifestyle, including diet, tobacco and alcohol consumption, obesity and physical inactivity, and environmental factors, such as exposure to chemicals or ionizing radiation, may also increase the risk of advanced PCa (Figure 3) [2,5,14].

### 3.1. Unmodifiable Risk Factors: Ethnicity, Family History, and Genetic Factors

PCa is infamous for its ethnic disparity, which raises the possibility that inheritance plays an important role in oncogenesis. The highest incidences of this cancer are documented in descendants of Northern Europeans and African-Americans, while native Africans and Asians are much less susceptible to the disease [55]. For instance, African-American, Caribbean, and Black men in Europe have the highest incidences of PCa and are more likely to develop the disease earlier in life when compared to other racial and ethnic groups [17,56,57]. These individuals possess a common genetic background more prone to the development of cancer, such as specific genes (e.g., chromosome 8q24) that are more susceptible to mutation (Table 1) [2,16,58]. The migration and colonization history of Scandinavians is intimately related to the susceptibility to PCa in Europe. Subsequently, the incidences in other ethnic groups are related to the history of European settlement and the degree of admixture. Some research has suggested that PCa has been transmitted through a hereditary predisposition that resides in the Northern European genome [55]. A proportion of the patients in the European, European American, and African-American populations share two polymorphisms at chromosome 8q24, transmitted by admixture [59,60,61]. The low frequency of these alleles among native Africans and other ethnic groups, however, suggests transmission by admixture between Europeans and African-Americans. The Caribbean countries have a history of colonization by Europeans, including the Scandinavians. At the same time, the slave trade brought many Africans. Given these reasons, many of the Caribbean countries now show high PCa incidence [55].

The prevalence of family PCa is estimated to be around 20%, while the rate of inherited PCa is about 5% to 15% [10,21]. The presence of similar genes, similar lifestyles, and similar environmental conditions are among the reasons associated with family PCa. Inherited PCa occurs when a gene mutation is transmitted from one generation to the next, when at least three of their first-degree relatives are affected by PCa, or when three or two generations of a family, or more close relatives (such as the father, brother, son, grandfather, uncle, or nephew), are affected by this cancer [21,22]. Some cancer predisposition genes have been identified to affect the risk of PCa, including hereditary mutation of *HOXB13* as well as *BRCA1*, *BRCA2*, *ATM*, *CHEK2*, and *PALB2*, and Lynch syndrome *MLH1*, *MSH2*, *MSH6*, and *PMS2* genes (Table 1) [21]. Other genes have a poorly defined cancer risk with unknown clinical significance. Nevertheless, the genetics behind family and hereditary PCa remains complex [10,21,22].

### 3.2. Modifiable Risk Factors: Lifestyle, Diet, and Environment

Lifestyle factors are modifiable and may provide an effective method for reducing cancer risk (Figure 3). According to the World Health Organization (WHO), 30 to 50% of cancers are preventable by healthy lifestyle choices, such as avoidance of tobacco and alcohol consumption, and public health measures, such as immunization against cancer-causing infections [3,5,14,32]. Men with PCa have been shown to exhibit upregulated oxidative stress and impaired antioxidant defense systems [62]. Animal studies have reported that nutrients, such as fat, protein, carbohydrates, vitamins (vitamins A, D and E), and polyphenols, are involved in PCa pathogenesis, and progression through several mechanisms, including inflammation, antioxidant effects, and the effects of sex hormones [63]. However, it has been difficult to determine which nutrients have a beneficial or harmful impact on PCa incidence and progression due to divergent results in clinical studies [3,32,64].

Diets involving plant-based foods, such as tomatoes, cruciferous, and soybeans, have been associated with a lower risk of developing PCa [32,43,49]. Cruciferous or Brassica vegetables are known to possess anticancer properties mediated by phenylethyl isothiocyanate, sulforaphane, phytochemicals, and indole-3-carbinol [54]. Similarly, lycopene, a carotenoid mostly found in tomatoes and other red fruits and vegetables, has been shown to have powerful antioxidant properties and cancer-preventive effects by reducing lipid peroxidation and inhibiting cell growth [39,40,41,65], and is associated with a decreased risk of PCa [41,42,44]. Such effects are certainly correlated with the observation that lycopene acts on the androgen receptors and reverses the effects of dihydrotestosterone [66]. Soy and green tea have also been investigated for their chemo-preventive capacity in relation to PCa (Table 1). Soy isoflavones and their derivatives, genistein and daidzein, reportedly show efficacy in preventing PCa [63]. Genistein acts as a chemotherapeutic agent in various cancer cells, modulating cell angiogenesis, apoptosis, and metastasis [62]. Moreover, soy isoflavones are similar in structure to 17β-estradiol, and thus can bind to the estrogen receptor and act as phytoestrogens. In addition to estrogenic effects, isoflavones reportedly exert antioxidant and inhibitory effects on tyrosine kinase activity [63]. However, the inadequate intake of isoflavones may lead to PCa progression [63]. The catechins found in green tea exhibit anticarcinogenic effects that may prevent various stages of carcinogenesis and metastasis [50,51,52,53]. Vitamin D and its analogues seem to protect from PCa, through the inhibition of cell proliferation and invasion, and inflammatory signaling (Table 1). For instance, several epidemiological studies suggest that PCa occurs more frequently in older men with vitamin D deficiency [2,47,67]. Moreover, a high dietary intake of dairy products rich in calcium, higher than the daily recommendation, also increases PCa risk, due to decreased serum levels of vitamin D [45,46,48,68]. Nevertheless, the research about nutrient intake and PCa needs to be further elucidated and extended.

Several epidemiological studies have shown a positive correlation between PCa mortality and per capita consumption of meat, fat, and dairy products [3,32,33,65]. The promotion of prostate carcinogenesis through androgen signaling, increased levels of reactive oxygen species (ROS), leukotrienes, and prostaglandins from lipid metabolism, as well as increased basal metabolism, IGFs levels, and tumor proliferation, are a few biological mechanisms that are thought to connect trans and saturated animal fat and PCa risk. Additionally, aromatic hydrocarbons and mutagenic heterocyclic amines, which are formed while cooking all of the components in meat at high temperatures—including creatine, amino acids, and sugar—can result in lipid peroxidation and DNA damage through the production of free radicals [2,69]. Unsaturated fatty acids such as Omega-3 fats, abundant in fish and vegetable oils, have been reported to reduce the risk of PCa. However, Omega-6 fats seem to have a pro-inflammatory effect through linoleic acid [2,70]. Arachidonic acid, a metabolite of linoleic acid, leads to the formation of pro-inflammatory prostaglandins (PG), such as PGE2, involved in cell proliferation, and 5-hydroxyeicosatetraenoic acid, which is found to be increasingly expressed in malignant PCa [3,32,33].

Changes in the metabolic profile caused by metabolic disorders such as obesity, insulin resistance, and changes in the hormonal profile are often associated with PCa, and some conditions can lead to more aggressive tumors [3,32,33,34]. Obese men show alterations in circulating levels of metabolic and sex steroid hormones, both known to be involved in prostate development and oncogenesis. Clinical studies have demonstrated that obesity might have clinical implications for disease detection and management [27,28,71]. Additionally, insulin is a risk factor of promoting PCa initiation and/or progression. In aggressive PCa tumors, for instance, elevated circulating insulin concentrations were found, supporting the role of insulin in PCa growth [72]. Tobacco consumption is another PCa risk factor (Table 1) [34,36,37]. The incidence and mortality rates of PCa have increased significantly with the increase in tobacco use, due to exposure to carcinogens and alterations in circulating levels of hormones [73]. Functional polymorphisms in genes involved in the polycyclic aromatic hydrocarbons (PAHs) metabolism, one of the carcinogenic chemicals of cigarette smoke, may affect cancer onset and progression [2]. Researchers found that smoking increases the metabolism of serum estrogen, which is involved in a more aggressive tumor phenotype, resulting in increased PCa-related deaths [74]. Moreover, cigarette smoking has been associated with adverse pathological features and worse oncological control [10].

## 4. Prostate Cancer Screening

**Figure 4:**
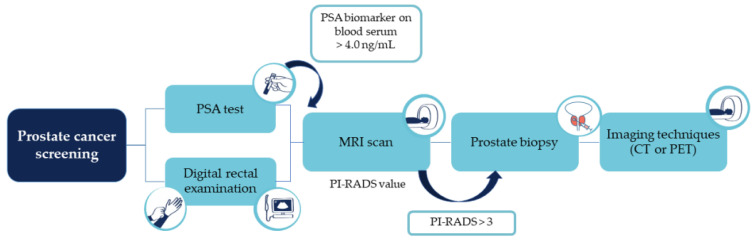
PCa diagnosis pathway.

Screening for PCa is based on the PSA biomarker values in blood serum (>4.0 ng/mL) and DRE. After suspicion, a magnetic resonance imaging (MRI) scan is usually performed, which indicates whether a prostate biopsy should be performed, considering the prostate imaging–reporting and data system (PI-RADS) value (PI-RADS > 3). Following the histological confirmation (biopsy) of malignant neoplasia, staging tests are performed, through imaging techniques such as computed tomography (CT) or positron emission tomography (PET). In turn, the results of these tests dictate the patient’s therapy based on a combination of surgical strategies, hormone therapy, radiotherapy, and chemotherapy (Figure 4) [75].

PSA is a glycoprotein normally expressed by the prostate tissue with a cut-off of 4.0 ng/mL [5]. However, this test shows low selectivity to detect PCa and monitor the disease’s progression [76], due to its limited sensitivity (20.5%) [77], accuracy (62–75%) [78], and specificity (51–91%) [79]. PSA screening cannot differentiate patients in terms of the aggressiveness of the tumor [80], and cannot distinguish between benign prostatic hyperplasia and prostatitis [81]. Furthermore, PSA levels may be affected by several other factors, such as age, body mass index (BMI), and urinary tract infection, leading to false-positive results [77,82]. Due to concerns about overdiagnosis and overtreatment, along with the high rate of false-positive results, the United States Preventive Services Task Force made recommendations against PSA testing among men over 70 years old [7,76]. This decision resulted in a decline in the incidence of PCa from 2007 to 2014. Between 2013 and 2017, the mortality rates flattened, most likely because of a decline in the use of PSA, which consequently resulted in the diagnosis of more men with metastatic PCa [76]. Therefore, it has become very important that men are fully informed of the potential benefits and harms of PSA screening [83].

A decisive diagnosis of PCa is based on a prostate biopsy when PSA and DRE show abnormal results [84,85]. Besides being an invasive, unpleasant, and potentially harmful procedure [86], prostate biopsies also show the risk of severe infection, due to the introduction of rectal commensal or other bacteria through a needle into the sterile prostate [87]. Moreover, this procedure can still lead to both false-positive and false-negative results [2,88,89]. False-negative results may occur when the tumor is small, when the cancer cells are distributed heterogeneously, and in early PCa stages when, histologically, the tumor appears benign. Accordingly, the samples obtained during the biopsy may not be representative of cancer. Another issue is the overdiagnosis and overtreatment of relatively indolent tumors with low potential for morbidity or death if left untreated [90,91]. Hence, serum PSA levels and prostate biopsy histology have very limited accuracy in predicting the clinical behavior of individual tumors, especially the ones prone to becoming aggressive at a later stage. Several studies have focused on the development of new methods to overcome these limitations and provide more accurate tools for PCa detection and management (Table 2).

### 4.1. Prostate-Specific Membrane Antigen: A Theranostic Approach

Imaging methods are used to define the stage of PCa and so guide its management. However, PCa’s more aggressive forms can manifest rapid growth with progression to adjacent organs and spread to lymph nodes and bones [2,113,114], and CT, bone scan, and MRI have limited performance abilities in the detection of lymph node metastasis [92]. Patients with castration-resistant PCa (CRPCa) have a 90 to 95% probability of developing bone metastases, which leads to severe morbidity, including bone pain, pathological fractures, spinal cord compression, and hematological consequences of bone marrow infiltration [115,116,117]. Due to the importance of bone metastases in the overall disease progression, bone-targeted therapy constitutes an essential part of the treatment of CRPCa [118]. A possible therapy may be based on the use of radiopharmaceuticals systemically administered to slow or reverse the bone metastatic progression [117].

Current research is focused on the molecular targeting of prostate-specific membrane antigen (PSMA) as a theragnostic approach, to diagnose, monitor, and treat PCa [92]. PMSA is a transmembrane enzymatic protein found on most PCa cells, and its overexpression correlates to adverse factors, such as androgen independence, metastasis, and progression, making PSMA an antigenic marker for PCa progression [92,93,117,118]. Hence, PMSA can be used for diagnostic and therapeutic purposes, and several clinical trials have been investigating its effectiveness as a diagnostic tool and for direct radioligand therapy (Table 2) [92].

#### 4.1.1. Molecular Imaging

PSMA scans can detect metastatic lesions that are missed by conventional imaging techniques [92], so small molecules, antibodies, and antibody fragments that target PSMA have been created, radiolabeled, and used for molecular imaging [98].

PET is emerging as a highly sensitive molecular imaging technique in the detection and localization of primary PCa. PET uses a positron emitter to label key molecules that are intravenously injected, and their distribution and uptake images provide insights into metabolic changes associated with cancer [119]. This technique has been reported as a valuable tool in the diagnosis of PCa patients with negative MRI and systematic biopsies [98]. Recently, ligands of PSMA were introduced in PET to diagnose and manage PCa (reviewed by Mena et al., 2020 [99]). This approach can improve PCa detection by identifying lesions that are not visible on MRI, providing better estimates of tumor volume [98]. PSMA-PET can be used in the diagnosis, staging, and management of PCa patients [99]. PSMA-PET has an important role in the initial staging of PCa, superior diagnostic performance to anatomical imaging, and enhanced sensitivity to detect node metastasis (reaching 99% [119]), outperforming other molecule imaging techniques, including PET-CT [98,99]. Furthermore, PSMA-PET can be combined with anatomical CT (PET/CT) and MRI (PET/MRI) images for the detection of bone metastases [99,100] (Table 2). PSMA-PET/MRI consistently outperforms multiparametric MRI (mpMRI) in the detection or localization of PCa in intermediate- or high-risk PCa patients (reviewed by Moradi et al., 2021 [98] and Mena et al., 2020 [99]). PSMA-PET/CT has greater sensitivity in the detection of bone metastasis when compared to whole-body bone scans [100], and has shown the most utility in biochemical recurrence [119]. PSMA-PET/CT was first coupled with gallium-68 (^68^Ga) and is considered the most sensitive and specific method for staging high-risk PCa and imaging recurrent PCa [92,98]. Moreover, ^68^Ga-labeled ligands have shown higher sensitivity and specificity in the diagnosis of primary and recurrent PCa [100]. In a retrospective analysis, Maurer et al. [120] investigated the diagnostic efficacy of ^68^Ga-PSMA-PET for lymph node staging in patients with PCa and compared it to CT and MRI imaging. In their analyses, ^68^Ga-PSMA-11 showed sensitivity, specificity, and accuracy levels of 65.9%, 98.9%, and 88.5%, respectively, in the detection of nodal metastases, compared with the values of 43.9%, 85.4%, and 72.3% achieved by morphological imaging [120]. In another study, Thomas et al. [100] investigated the difference between technetium-99m (^99m^Tc)-methyl diphosphate (MDP) bone scans and ^68^Ga-PSMA-PET/CT for the detection of bone metastases in PCa. The authors compared the number of identified lesions and found that the PSMA-PET/CT method detected twice the number of lesions, especially in the thorax and pelvis. Their results suggest that when patients go through ^68^Ga-PSMA-PET/CT, the bone scan is not mandatory [100].

#### 4.1.2. Radioligand Targeted Therapy

Recent studies suggest that newer molecular theragnostic approaches, based on PSMA radioligands, have the potential to provide even more effective and personalized treatment options for diagnostic, prognostic, and therapeutic applications in patients with CRPCa, with fewer toxicities and adverse effects [92,93,94]. This approach has been developed to select patients, and delivers irradiation to all tumor sites, including osseous, nodal, and visceral metastases [92]. PSMA radioligand therapy uses small-molecule inhibitors of PSMA, usually labeled with beta and alpha-emitting radionuclides that emit cytotoxic radioactive decay [92,93]. Alpha and beta radionuclides differ in energy, tissue range, linear energy transfer, and the number of DNA hits needed for cell destruction [117]. These radiopharmaceuticals deliver targeted irradiation to the active bone turnover sites, where metastatic infiltration and destruction are happening. This approach can simultaneously treat multiple sites of disease, ease administration, and be integrated or combined with other treatments. Alpha-emitters include actinium-225 (^225^Ac), thorium-227 (^227^Th), radium-223 (^223^Ra), and astatine-211 (^211^At). Recently, ^223^Ra was approved to treat bone metastases from PCa. This authorization follows the symptomatic relief and significant improvement in the overall survival of CRPCa with predominant bone metastases that ^223^Ra was shown to elicit [121]. Beta-emitting radiopharmaceuticals, including lutetium-117 (^177^Lu), strontium-89 (^89^Sr), samarium-153 (^153^Sm), and rhenium-186 (^186^Re), have been used for bone palliation. ^177^Lu is the most used beta-emitter, due to its favorable safety profile, short range of emissions, and relatively long half-life, allowing the delivery of a high degree of radiation to specific lesions [92]. For instance, [^177^Lu] Lu-PSMA-617 shows a favorable safety profile due to reduced kidney uptake, and has demonstrated promising results in prospective trials with high response rates, low toxic effects, and the reduction of pain in men with metastatic CRPCa who progressed after standard treatments [95,96,97]. In general, radioligand therapy shows promising response rates with low toxicity in extensively pretreated patients with PCa [92]. While most of these studies remain experimental and the effects of this therapy on overall survival and safety are yet to be determined, their clinical observations are very promising [95,118,122,123,124].

PSMA-targeted imaging and therapy have proven to be excellent diagnostic and therapeutic options for metastatic PCa, but further studies are still required to determine the effect of this approach on overall survival and safety. Moreover, current research is still ongoing regarding the exact role of PSMA in various stages of PCa care [92].

### 4.2. Tumor Biomarkers

In recent years, new potential biomarkers for PCa screening and management have been developed through advances in molecular medicine, particularly OMICs genomics, proteomics, transcriptomics, and lipidomics. In addition to molecular biomarkers for urine, serum, and tissue samples, extracellular vesicles (EVs), circulating tumor cells (CTCs) and DNA (ctDNA), and cell-free DNA (cfDNA), common liquid biopsy biomarkers [125] and long noncoding ribonucleic acids (lncRNAs) have emerged as promising PCa biomarkers.

#### 4.2.1. Molecular Biomarkers

Based on the combination of imaging techniques with other methodologies such as gene or protein profiling, several molecular biomarkers have been developed for urine, serum, and tissue samples to improve cancer detection, pre-biopsy decision-making, cancer risk assessment, and the therapeutic management of PCa [126]. Additionally, risk calculators (RCs) are used in combination with these tests to help identify each individual’s specific cancer risk, hence reducing the number of unnecessary biopsies. The guidelines on PCa treatment are therefore recommending the use of these tests in addition to the current PCa screening methods [77]. These biomarkers include several derivatives of PSA, such as the Prostate Health Index (PHI), approved by the US Food and Drug Administration (FDA), which combines total PSA, free PSA, and [−2] proPSA, and the Four-Kallikrein (4KScore) blood tests, which consist of kallikrein-related peptidase 2 (hK2), intact PSA, free PSA, and total PSA [104]. Transcriptomic methodologies also contributed to the discovery of biomarkers, and Progensa Prostate Cancer Antigen 3 (PCA3) is the first and only urine test approved by the FDA, which detects the PCa gene 3 transcript levels. The MyProstateScore (MPS) assay requires the collection of urine post-DRE and is based on combinations of multiple gene analyses, including total serum PSA, the PCA3 assay, and the expression of the TMPRSS2: ERG fusion gene [127,128]. These biomarkers can be used in liquid biopsies and involve a combination of clinical information, including age, family history, DRE result, PSA levels, and prostate biopsy history, with genetic and epigenetic changes. Nevertheless, the technologies associated with these approaches are expensive and unavailable in many medical facilities. Other factors such as tumor heterogeneity, tumor–host interplay, complexity, multiplicity, and redundancy of tumor–cell signaling networks must be overcome to develop effective biomarkers [81].

#### 4.2.2. Long Non-Coding RNAs

LncRNAs are RNA transcripts that are longer than 200 nucleotides and do not encode proteins. LncRNAs have been found to exhibit abnormal expression in various types of cancer, including PCa. Most lncRNAs linked to PCa are overexpressed in tumor tissues and cancer cells, contributing to tumor proliferation, invasion, and metastasis. In turn, only a small number of lncRNAs are downregulated and may function as tumor suppressors in addition to their roles as transcriptional regulators and oncogenes [106]. All these unique features make lncRNAs promising prognostic biomarkers and therapeutic targets for the diagnosis, screening, prognosis, and progression of PCa [106] (Table 2). Recent research has demonstrated that lncRNAs such as PCA3, GAS5, and HOTAIR are associated with the development and progression of PCa [106]. Given its higher specificity and sensitivity than the PSA blood test, PCA3 is one of the most well-studied lncRNAs. Additionally, its combination with PSA testing or other biomarkers will significantly improve the sensitivity, specificity, and accuracy of PCa screening and diagnosis. For instance, the use of PCA3 in conjunction with TMPRSS2-ERG tests can reduce the number of unnecessary biopsies and increase diagnostic accuracy [106]. Another putative PCa diagnostic marker is MALAT1, whose increased expression has been linked to high PSA levels and Gleason scores, as well as with tumor stage and CRPCa [106]. Single-nucleotide polymorphisms of *MALAT1* were investigated by Hu et al. [109], who found that rs619586 and rs1194338 were significantly associated with PCa’s susceptibility to both advanced Gleason grade and nodal metastasis. A noninvasive post-DRE urine assay based on the combination of the lncRNAs PCA3 and MALAT1 for the early diagnosis of PCa and high-grade tumors was developed and validated by Li and collaborators [110]. However, according to some researchers, the PCA3 test is affected by intra-individual variability, being unable to differentiate between high-grade and low-grade tumors. Hence, more data are necessary to determine PCA3’s application in PCa diagnosis [106]. The lncRNAs TMPO-AS1 and FALEC have shown their potential utility as biomarkers for PCa diagnosis and progression [106,112]. Zhao et al. [108] examined the biological role of FALEC in PCa cell lines as well as its expression profile, and paired histologically normal tissues. In 85 patients, clinical PCa tissues showed significantly higher FALEC expressions when compared to adjacent normal tissues. Moreover, in vitro cell proliferation, migration, and invasion could be inhibited by the downregulation of FALEC. According to these findings, FALEC may be a useful diagnostic and therapeutic target in PCa patients [108]. Li et al. [107] investigated the expression, prognostic value, and functional role of lncRNA BDNF-AS in PCa. The authors also correlated the expression of BDNF-AS with the clinicopathological factors of patients. The results of this study demonstrate the potential use of BDNF-AS as a prognostic biomarker for PCa patients with poor prognoses and shorter overall survival, as it was downregulated in these cases. Furthermore, lncRNAs can be used to predict the recurrence of biochemical events. SChLAP1 was highly expressed in PCa tissue, which was substantially correlated with biochemical recurrence, clinical progression, and PCa-specific mortality [111]. Additionally, SChLAP1 can be easily detected in urine, an important feature for the development of an SChLAP1 assay for guided therapy (as reviewed by Xu et al. [106]). Given the roles of lncRNAs in PCa, it will be important to create specific drugs that interfere with malignant signaling networks in which lncRNAs are engaged, particularly in PCa cells. However, it is still unclear how exactly lncRNAs work at the molecular level, it being essential to further investigate the role of lncRNAs in prostate carcinogenesis [106].

#### 4.2.3. Liquid Biopsy Biomarkers

Liquid biopsy has emerged as a complement to invasive tissue biopsy to guide cancer diagnosis and treatment [76]. Liquid biopsies rely on the detection of specific biomarkers in readily accessible body fluids, such as blood, serum, or urine [89]. The common liquid biopsy biomarkers are EVs, CTCs, ctDNA, and cfDNA, which provide specific information based on their intrinsic characteristics. CTCs are cancer cells from primary and metastatic tumors that are released into the vasculature and circulate through the body to form metastatic niches in other tissues, being detectable in cancer patients only [125]. Similarly, ctDNA is a tumor-derived short, fragmented DNA found in the bloodstream, which reflects cancer-related genetic changes. cfDNA or RNA (cfRNA) are cell-free circulating small nucleic acid fragments that are released after the lysis of apoptotic or necrotic cells. cfDNA is detectable in blood and urine samples from patients with cancer, and their analyses improve the evaluation of mutations, polymorphism, methylation, and loss of DNA integrity [76,89,129]. Numerous studies have shown the relevance of liquid biopsies in PCa screening. cfDNA and EVs seem to have a better application in the diagnosis and prognosis of PCa than CTCs [76,87,89,101] (Table 2). This occurs because early-stage or localized PCa patients have very few CTCs and their use is more effective in the later stages of this cancer [89]. The only FDA-approved liquid biopsy test for PCa, CellSearch, is based on the detection of CTCs, and there is no evidence of the wide clinical implementation of this technology in medical practice. EVs are nano-sized, double-lipid membrane vesicles, such as exosomes and microvesicles, that are secreted from cells and shed into biofluids, including blood and urine [104]. EVs are involved in intercellular communication and immune function, through proteins, lipids, mRNA, microRNAs (miRNAs), and DNA, and have been correlated to the presence of cancer for diagnostic purposes (Table 2) [76,101,130,131]. Cells exchange proteins, nucleic acids, sugars, and lipids through EVs to induce changes in the recipient cells, which makes EVs potential carriers of cancer biomarkers from tumor cells to other tumor or non-tumor cells [89]. EVs can also be used as a vehicle for drugs or nucleic acids with antineoplastic effects [87,102]. The EVs approach may improve the sensitivity of PCa biomarkers, given the protective role of the EVs’ lipid layer over biomolecules, meaning that the concentration of PCa biomarkers will be higher in EVs [89]. Urine is the most used body fluid for the detection of biomarkers in EVs from liquid biopsies of PCa. Moreover, exosomal miRNAs are emerging as promising prognostic biomarkers for metastatic CRPCa patients [89]. The concentration of RNA-based biomarkers, particularly miRNA, is higher in EVs than in CTCs from urine samples. Nevertheless, the application of miRNA as a diagnostic marker has been limited due to a lack of specificity, and in turn, many studies have emerged to investigate EV-mRNA as a diagnostic and prognostic biomarker for PCa management [76]. McKiernan et al. [104] developed an exosome-derived gene expression signature from normalized PCA3 and ERG RNA from urine predictive of initial biopsy results. Exosomes in post-DRE urine of PCa patients contain both PCA3 and TMPRSS2: ERG mRNA. In their study, the authors were able to develop a molecular signature predictive of PCa combined with serum PSA in a diagnostic test, which was able to discriminate between benign disease and high- and low-grade tumors, reducing the total number of unnecessary biopsies [104]. Ji et al. [105] developed a strategy for exosomal mRNA detection based on features of mRNA of circulating exosomes and identified a PCa exosomal mRNA signature for PCa screening and diagnosis. With this strategy, the authors were able to distinguish PCa patients from healthy controls [105]. Despite the beneficial properties of EVs for the diagnosis of PCa, their clinical application still presents a few challenging issues [76]. EVs are released from all cells in the body, which makes it difficult to determine which EVs are tumor-derived, meaning that new technologies for the specific detection and isolation of tumor-derived EVs need to be developed [76]. Recent EVs isolation technologies have been developed to improve isolation performance, yield, purity, usability, hands-on procedures, and processing time [76]. However, EVs isolation is still difficult, especially in EVs from blood plasma, due to the purity and efficiency achieved by laboratory procedures. Moreover, there is no wide clinical application of liquid biopsies of PCa with EVs [89], and automated analysis platforms are yet to be developed for large-scale clinical studies [76]. Overall, the use of CTCs and EVs as biomarkers of PCa in liquid biopsies is being hindered by some issues, such as the inexistence of specific guidelines for the biomarker’s isolation and detection. Additionally, the validation and standardization of the microfluidic devices used in liquid biopsies has not been achieved yet [129].

### 4.3. Active Surveillance and Risk-Stratification Algorithms

PCa is very heterogeneous in terms of grade, phenotypes, and genetics, displaying complex features [2]. This tumor often has indolent growth, which does not compromise the patient’s quality of life, but its diagnosis and subsequent treatments have a high impact on the physical and mental status of patients, significantly affecting their quality of life [81]. The main goal of early detection is to identify PCa in a phase whereat it needs less aggressive treatments with fewer side effects and has a higher chance of cure, even in the cases of locally advanced and metastatic PCa. Many early diagnoses can be safely managed by active surveillance, preventing overtreatment, thereby improving or maintaining the patient’s quality of life and avoiding adverse outcomes [132].

Active surveillance consists of the serial monitoring of disease progression, through PSA tests, DRE, and biopsies, to track cancer growth. This has become the preferred approach for men with low-grade PCa [2,133], as men can avoid immediate treatment and prospective side effects [2]. When discussing therapy choices and in the selection criteria for active surveillance programs [134], external factors, such as obesity, BMI, and the hormonal profile (e.g., testosterone levels), should be considered by the clinical practice, since all these factors influence the PSA levels [135,136]. Recent studies suggest that the conjugation of PSA screening with other methodologies, such as risk RCs, biomarkers, and imaging techniques such as MRI, can attenuate overdiagnosis and underdetection issues [137]. Van Poppel et al. [137] proposed a risk-stratified algorithm, combining MRI, RC, and PSA tests, that improves the efficiency of “PSA-only” screening and reduces unnecessary biopsies and overdiagnosis. The combination of these tools improves the individual balance between the harms and benefits of early detection in well-informed men who are at risk of having PCa [137]. Based on the initial PSA test result and age, different time intervals for repeated PSA testing are proposed, reflecting the likelihood of a future diagnosis of clinically significant cancer. This strategy helps to avoid false-positive biopsies, as low-risk men can go through individualized PSA tests and, if necessary, repeated MRIs to track cancer growth. Then, RCs seem to be the most appropriate approach to assessing the risk of developing PCa after PSA testing. RCs are accessible to every clinician, easy to use, inexpensive, and non-invasive. Moreover, MRI results can be integrated into an RC that includes PSA density as a continuous variable, to determine the need for a prostate biopsy in men with intermediate- and high-risk [137,138]. PSA density has been described to improve the specificity of the PSA test [138,139]. It is defined as the level of serum PSA divided by the prostate volume and presents a cut-off of 0.15 ng/mL^2^ [139]. PSA density can be used as a prognostic biomarker to determine which patients need to undergo definitive therapy from those who may be managed by active surveillance, as well as patients with a previously negative MRI who should proceed to a prostate biopsy [139]. This allows the more accurate evaluation of individual risk, which is essential for properly interpreting the MRI results. Consequently, only men who present a high risk of clinically significant PCa, according to an RC, will be proposed for a systemic biopsy after MRI [137].

Evidence shows that performing an MRI before a biopsy allows one-third of men to avoid an immediate biopsy and reduces overdiagnosis, with 40% fewer clinically unimportant cancers and approximately 15% more clinically significant cancers detected [137,140]. However, the implementation of MRI in the risk assessment of PCa is not yet fully realized in the whole of Europe [137], which in turn reflects the geographical differences in the incidence rates between European countries. To further reduce unnecessary biopsy procedures, the decision process of a biopsy in men with a PI-RADS of 3 should be carefully examined. The PI-RADS classification is based on a scale of values from 1 to 5, and determines the likelihood of clinically significant PCa. While PI-RADS values of 4 and 5 indicate that a biopsy is required, it is challenging to establish whether a biopsy should be performed or not in patients with a score of 3 [141]. Additionally, the PI-RADS score does not measure PCa aggressiveness, meaning that a biopsy is still needed. Research has found that excluding men with PI-RADS 1–2 or PI-RADS 3 lesions based on a low PSA density only increases the likelihood that clinically significant tumors will be undiagnosed due to nonvisual PCa or misinterpretation of the reader [137]. The European Association of Urology (EAU) guidelines strongly recommend performing an mpMRI before a biopsy to modify the management approach accordingly. This imaging approach presents preferable detection rates and reduces the number of biopsy procedures, particularly when MRI-negative men are excluded from prostate biopsy, due to its capacity to differentiate between significant and insignificant tumors [132]. Furthermore, the PI-RADS guidelines have recommended systematized mpMRI acquisition and the global standardization of reporting. Nevertheless, there is a lack of consensus on detailed aspects of mpMRI acquisition protocols [141].

Artificial intelligence (AI) methods have been proposed for a wide range of applications in the PCa diagnostic pathway [137,141,142,143]. AI can be used to improve the initial evaluation of prostate mpMRI cases and the image quality, as well as the detection and differentiation of clinically significant from insignificant cancers on a voxel level, and the classification of entire lesions into PI-RADS categories (reviewed by Belue and Turkbey [142] and Sunoqrot et al. [143]). Studies on MRI AI have revealed the role of AI in improving the clinical management of localized PCa, the interpretation of MRI and the data processing for biopsies, by reducing inter-reader variation and supporting the radiological workflow [142]. Nevertheless, AI requires caution in its use, as the proficiency of this method is still below that of an expert [141]. Moreover, more prospective studies with multicenter designs are required to understand the impact of AI on improving radiologists’ performance and the clinical management of PCa [137,142].

### 4.4. Volatilomics

**Figure 5:**
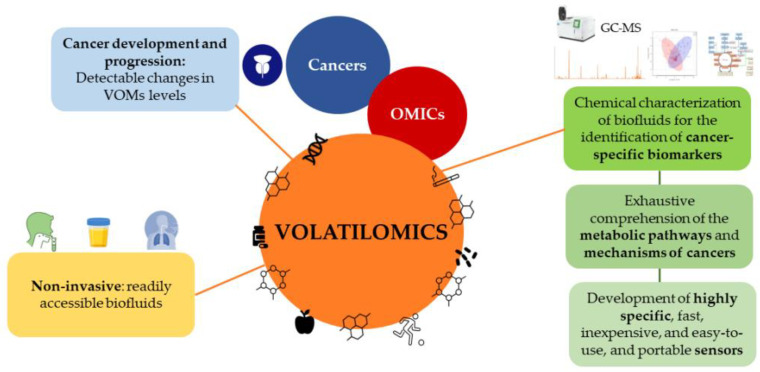
Cancer development and progression can lead to changes in the levels of volatile organic metabolites, which can be used to define a volatilomic biosignature for diagnostic purposes.

Emerging studies demonstrate that combining PSA screening with other methodologies, such as RCs, biomarkers, and imaging tests, e.g., MRI or fusion biopsies, might attenuate overdiagnosis and underdetection, eventually reducing the number of unnecessary biopsies [137]. Volatilomics, a subset of metabolomics, has recently emerged as a simple, effective, and non-invasive method with great potential for cancer screening. Volatilomics focuses on volatile organic metabolites (VOMs), which are low-molecular weight metabolites (<500 Da) with high volatility and a carbon-based chemical group [144]. VOMs are present in readily accessible biofluids, including saliva, urine, and exhaled breath, as they are produced by the metabolism of cells, reflecting their biological activity [145]. The progressive accumulation of genetic, epigenetic, and post-translational changes that support cancer growth can lead to changes in VOMs levels and, as a result, affect an individual’s volatilomic profile (Figure 5). Hence, VOMs are a rich source of data on health, since they can reflect the metabolic and biochemical alterations triggered by cancer progression. From this perspective, a volatilomic biosignature for diagnostic purposes can be defined using these changes [77,86].

Even though the volatilomics approach is relatively recent in PCa compared to other cancers [77,88,146,147], empirical data have confirmed its potential use in cancer screening, the monitoring of disease progression and effectiveness of treatment, as well as for the discrimination between different cancer types [86,148,149,150]. Different approaches involving volatilomic studies have been proposed to establish connections between cancer and the body’s VOMs signature using highly sensitive analytical techniques. In these studies, biofluids are chemically characterized to identify cancer-specific biomarkers using mass spectrometry-based techniques combined with multivariate statistical analysis. Another approach includes the identification of cancer-characteristic odor fingerprints through electronic noses (e-noses) [151]. However, since several VOMs have been suggested as PCa biomarkers and contradictory results on the same metabolites have emerged from different reports, it is difficult to establish reliable biomarkers, and no exhaustive studies have yet been published [151,152]. Additionally, a few restrictions hinder the implementation of these approaches in real-time diagnostic applications, and consequently, in clinical practice (reviewed by Berenguer et al. [144]). For instance, the ability to compare the outcomes of various studies between different laboratories is hampered by variations in sample preparation, analytical procedures, and statistical platforms [88]. Hence, methods must be standardized from sample collection to data processing, as well as assess the impact of confounding factors, such as epigenetics, diet, medication, genetics, and environmental exposure. Epigenetic factors play an important role in determining the clinical phenotypes of PCa. Therefore, due to genetic, environmental, and toxicological factors, as well as the different dietary habits around the world and their influence on the development of cancer, the volatilomic biosignatures and potential biomarkers will differ according to the region of the world [77,88,144,146,147].

Despite these limitations, volatilomics offers a wealth of informational potential that will allow a thorough understanding of the metabolic pathways, and a clarification of the mechanisms of cancers and how they impact the generation of VOMs [153]. Further analysis of the VOMs’ origin and a more accurate assessment of the impact of confounding factors on the volatilomic profile will be possible as a result of these findings [147]. Additionally, the definition of cancer biomarkers will be made possible through the detection and quantification of specific metabolites due to the standardization of procedures and the creation of highly focused sensors. These findings will foster the development of highly specific, fast, inexpensive, easy-to-use, and portable sensors that can be implemented in clinical practice [145,154], demonstrating the importance of the volatilomics approach [151,155]. Hopefully, the progress in volatilomics studies will unveil biomarkers suitable for the diagnosis of PCa, to be used as a supplement to the current approaches for the classification and screening of cancer [129], with possible applications in the active surveillance of patients and individualized care [81,144].

## 5. Conclusions

PCa is the second leading cause of oncological death worldwide. Changes in the metabolic profile caused by metabolic disorders such as obesity are often associated with PCa, and some conditions can lead to more aggressive tumors. Lifestyle factors are modifiable and may provide an effective method for reducing PCa risk. Nevertheless, the research into nutrient intake and PCa needs to be further elucidated to understand how men can change their dietary habits to prevent cancer growth. The current screening methods are invasive and have a low sensitivity to detect PCa, leading to overdiagnosis and overtreatment. Several studies have focused on the development of new methods to overcome these limitations and provide more accurate tools for PCa detection and management. Moreover, the development of testing strategies to maintain most of the benefits of screening, while reducing the harms, has become an important need. These strategies focus on the diagnosis of potentially fatal cancers at a point where treatment is still effective, while not involving the treatment of indolent cancers, saving patients and healthcare systems from the burden of unnecessary, invasive, and costly medical procedures [83]. Furthermore, the combination of the PSA test with different techniques for the diagnosis of PCa, such as MRI, RCs, and biomarkers, has been proposed to obtain a more effective stratification of the patients and provide more personalized treatment.

## Figures and Tables

**Table 1:** Prostate cancer risk factors and their roles in the development of this tumor (articles from the last 5 years).

Risk Factor	Role in PCa	Reference
Ethnicity	PCa incidence, morbidity, and mortality rates vary significantly by race and ethnicity. African-American, Black, and Caribbean men show the highest PCa rates worldwide. These disparities are mostly related to differences in access to screening and treatment, exposure to PCa risk factors, and variations in genomic susceptibility (e.g., risk loci found at chromosome 8q24), among other biological factors.	[15,16,17,18,19,20]
Family history and genetic factors	According to estimates, around 5 to 15% of PCa cases have been related to hereditary factors. In genome-wide association studies, almost 170 loci of susceptibility for hereditary PCa (about 33% of familial PCa risks) have been identified. Many genes show a strong association with hereditary PCa risk, including *BRCA1*, *BRCA2*, *ATM*, *CHEK2*, and *PALB2*, and Lynch syndrome *MLH1*, *MSH2*, *MSH6*, and *PMS2* genes. Other genes, however, have an unclear cancer risk and unknown clinical importance.	[4,20,21,22,23,24,25,26]
Obesity, overweight and physical inactivity	Obesity is implicated in the dysregulation of various hormonal pathways, leading to higher levels of insulin and IGF, oxidative stress, and inflammatory cytokines, and lower levels of adiponectin, testosterone, and sex hormone-binding globulin. Obesity is associated with an increased risk of PCa mortality and recurrence, worsened treatment-related adverse effects, development of obesity-related comorbidities, and the earlier progression and development of metastatic disease. Nevertheless, the physiological mechanisms associated between obesity and poor PCa outcomes remain unknown.	[3,27,28,29,30,31,32,33]
Tobacco use	Smoking increases the risk of death from PCa, which increases with obesity, specifically for advanced PCa. Moreover, tobacco smoking increases the risk of biochemical recurrence and metastasis. Nevertheless, the association between tobacco smoking and PCa prognosis needs to be explored.	[3,32,34,35,36,37,38]
Lycopene and tomato-based products	Epidemiologic studies have focused on tomatoes as a specific source of lycopene, with more consistent findings supporting the protective effect of a higher intake of tomatoes on PCa risk. Furthermore, studies have shown a reduced risk of advanced PCa with the consumption of cooked tomatoes, since these products have more available lycopene. Current epidemiologic evidence is not definitive but suggests that a higher intake of tomato-based products is associated with a reduced risk of PCa and a potentially lower risk of progression. Further studies are required to determine whether the effect is because of lycopene or other components of tomatoes.	[3,32,39,40,41,42,43,44]
Calcium, dairy products, and vitamin D	An intake of dairy products above the daily recommended dose has been positively associated with PCa risk. A potential mechanism underlying the association with calcium is through suppressing circulating levels of dihydroxyvitamin D, which seems to have a protective effect against PCa. The mechanisms behind this association are not yet fully understood, but researchers suggest reducing dairy intake while increasing the consumption of fish and tomato products for PCa prevention.	[3,32,45,46,47,48]
Cruciferous, soy, and green tea	Cruciferous, soy, and green tea seem to have a role in decreasing the risk of PCa due to compounds with anticarcinogenic properties in their composition. Asian populations consume soy foods as a part of their regular diet, which might contribute to the lower PCa incidence found in these countries. However, the preventive action of these compounds needs to be further explored.	[32,43,49,50,51,52,53,54]

**Table 2:** Emerging diagnostic methods for prostate cancer detection and management (articles from the last 5 years).

Method	Evidence/Aim	Reference
PSMA radioligand targeted therapy and molecular imaging	Evidence: Molecular imaging techniques detect PCa lesions that are occult on anatomic imaging. PSMA radioligand therapy shows promising response rates with low toxicity in extensively pre-treated patients with PCa. Aim: Theragnostic applications—diagnosis, management, and treatment of metastatic PCa.	[92,93,94,95,96,97,98,99,100]
EVs	Evidence: EVs can mediate PCa progression and metastasis. EVs have great potential to be used as liquid biopsy biomarkers in the diagnosis of PCa. EVs can be used in risk stratification and to predict the response to hormonal, chemo-, immune- and targeted therapy. Aim: Diagnosis and treatment. Can be used to personalize and guide treatment decisions.	[76,87,89,101,102,103,104,105]
lncRNAs (*PCA3*, *MALAT1*, *SChLAP1*, *BDNF-AS*, *FALEC*)	Evidence: lncRNAs provide new insights into cancer signaling networks, along with novel strategies and methods for PCa diagnosis and treatment. lncRNAs analysis has the potential to improve the specificity and sensitivity of existing biomarkers. Aim: Novel biomarkers (predictive, diagnostic, prognostic) and therapeutic targets.	[106,107,108,109,110,111,112]

Legend: EVs: extracellular vesicles; lncRNAs: long non-coding RNAs; PSMA: molecular targeting of prostate-specific membrane antigen.

## Data Availability

Not applicable.
